# Comprehensive Analysis of mRNA and lncRNA Transcriptomes Reveals the Differentially Hypoxic Response of Preadipocytes During Adipogenesis

**DOI:** 10.3389/fgene.2020.00845

**Published:** 2020-08-06

**Authors:** Jinwei Zhang, Jideng Ma, Xiankun Zhou, Silu Hu, Liangpeng Ge, Jing Sun, Penghao Li, Keren Long, Long Jin, Qianzi Tang, Lingyan Liu, Xuewei Li, Surong Shuai, Mingzhou Li

**Affiliations:** ^1^Farm Animal Genetic Resource Exploration and Innovation Key Laboratory of Sichuan Province, Sichuan Agricultural University, Chengdu, China; ^2^Chongqing Academy of Animal Sciences, Chongqing, China; ^3^Key Laboratory of Pig Industry Sciences, Ministry of Agriculture, Chongqing, China; ^4^Chongqing Key Laboratory of Pig Industry Sciences, Chongqing, China; ^5^Jinxin Research Institute for Reproductive Medicine and Genetics, Chengdu Xi Nan Gynecological Hospital, Chengdu, China

**Keywords:** hypoxia, adipogenesis, mRNA, lncRNA, expression pattern

## Abstract

Local hypoxia has recently been reported to occur in the white adipose tissue (WAT) microenvironment during obesity. Adipocytes have a unique life cycle that reflects the different stages of adipogenesis in the WAT niche. Long non-coding RNAs (lncRNAs) play an important role in the cellular response to hypoxia. However, the differentially hypoxic responses of preadipocytes during adipogenesis and the potential role of lncRNAs in this process remain to be elucidated. Here, we evaluated the differentially hypoxic responses of primary hamster preadipocytes during adipogenesis and analyzed mRNA and lncRNA expression in same Ribo-Zero RNA-seq libraries. Hypoxia induced HIF-1α protein during adipogenesis and caused divergent changes of cell phenotypes. A total of 10,318 mRNAs were identified to be expressed in twenty libraries (five timepoints), and 3,198 differentially expressed mRNAs (DE mRNAs) were detected at five timepoints (hypoxia vs. normoxia). Functional enrichment analysis revealed the shared and specific hypoxia response pathways in the different stages of adipogenesis. Hypoxia differentially modulated the expression profile of adipose-associated genes, including adipokines, lipogenesis, lipolysis, hyperplasia, hypertrophy, inflammatory, and extracellular matrix. We also identified 4,296 lncRNAs that were expressed substantially and detected 1,431 DE lncRNAs at five timepoints. Two, 3, 5, 13, and 50 DE mRNAs at D0, D1, D3, D7, and D11, respectively, were highly correlated and locus-nearby DE lncRNAs and mainly involved in the cell cycle, vesicle-mediated transport, and mitochondrion organization. We identified 28 one-to-one lncRNA-mRNA pairs that might be closely related to adipocyte functions, such as *ENSCGRT00015041780*-*Hilpda*, *TU2105*-*Cdsn*, and *TU17588*-*Ltbp3*. These lncRNAs may represent the crucial regulation axis in the cellular response to hypoxia during adipogenesis. This study dissected the effects of hypoxia in the cell during adipogenesis, uncovered novel regulators potentially associated with WAT function, and may provide a new viewpoint for interpretation and treatment of obesity.

## Introduction

Obesity is a major public health issue in both developed and developing countries. It is a well-known risk factor for metabolism-related disorders including type II diabetes, cardiovascular diseases, and non-alcoholic fatty liver (Abarca-Gómez et al., 2017). White adipose tissue (WAT) is the largest calories storage depot and has an important role in maintaining systemic energy homeostasis. Obesity is accompanied by adipose tissue expansion that occurs through a combination of increasing adipocyte number (hyperplasia) and size (hypertrophy) (Rutkowski et al., 2015). Substantial evidence, especially from *in vivo* studies, indicates that WAT expansion induces local hypoxia that is similar to cancer (Ye et al., 2007; Lawler et al., 2016). Healthy hyperplastic WAT expansion is preferred for the adipose tissue response to excess energy intake. Local hypoxia synchronously leads to coordinated angiogenesis and extracellular matrix (ECM) remodeling that promotes *de novo* formation of adipocytes to encourage appropriate WAT expansion (Trayhurn, 2013). However, hypertrophic WAT expansion with uncoordinated angiogenesis will create local severe hypoxia and then triggers adipose tissue dysfunction (Crewe et al., 2017; Hammarstedt et al., 2018). Recent studies imply that an obesity-induced local hypoxic microenvironment in WAT may be involved in insulin resistance, inflammation, and fibrosis, which will further limit the expansion and lipid storage capacity of WAT and exacerbate obesity-associated metabolic disorders (Trayhurn, 2013; Foti and Brunetti, 2017).

The ubiquitous hypoxic response whether physiological [e.g., strenuous exercise (Ameln et al., 2005) and embryogenesis (Vasilev et al., 2016) or pathological [e.g., cancer (Bhandari et al., 2019) and ischemia (Koeppen et al., 2018)], whether external [organism level (Thomas et al., 2019)] or internal [cellular level (Eales et al., 2016)] has been a research hotspot in the past few decades. A decrease in oxygen partial pressure induces the hypoxic response of cells and initiates a series of adaptive programs that are mainly mediated by the family of hypoxia-inducible factors (HIFs), heterodimeric transcription factors containing an oxygen-dependent α subunit and constitutive β subunit (Semenza, 2012). During the cellular response to hypoxia, activated HIF-α dimerizes with HIF-β and then mediates the expression of HIF-regulated genes by binding to the hypoxia response element (5′-RCGTG-3′) of their promoter (Mylonis et al., 2019). HIF-regulated genes are mainly associated with multiple metabolically adaptive pathways, including angiogenesis, anaerobic metabolism, and erythropoiesis (Majmundar et al., 2010). Adipose tissue has high cellular heterogeneity, and adipocytes have a life cycle that reflects the different stages of adipogenesis (Jiang et al., 2012; Lynes and Tseng, 2018). Previous studies indicate that preadipocytes and mature adipocytes have differently hypoxic responses. Specifically, hypoxia inhibits preadipocyte adipogenesis by decreasing expression of PPAR-γ (Kim et al., 2005) and maintaining stem cell characteristics by upregulating Pref-1 (Moon et al., 2018). Hypoxia also induces a different secretome profile in preadipocytes and adipocytes, such as prompting leptin secretion in preadipocytes and increasing inflammatory adipokines and collagens in adipocytes (Wang et al., 2008; Rosenow et al., 2013). Moreover, lncRNA and miRNA patterns in preadipocytes and adipocytes show great differences (Kajimoto et al., 2006; Li et al., 2016). However, the hypoxic response of preadipocytes during adipogenesis has not been elucidated systematically.

**FIGURE 1:**
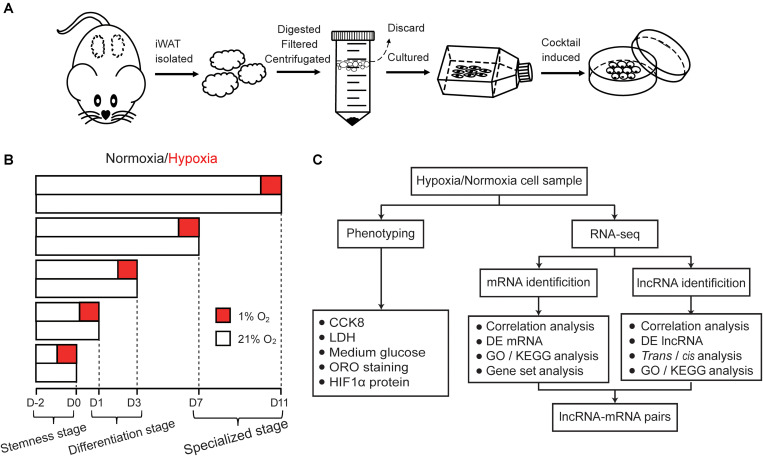
Schematic illustration of this study. **(A)** Schematic of hamster primary preadipocyte culture and differentiation. Firstly, iWAT were isolated and digested, then primary preadipocyte were obtained by filtration and centrifugation, finally, preadipocyte were differentiated by classic cocktail methods. **(B)** Schematic of hypoxic exposure, cell phenotype detection and cell sample collection during adipogenesis under normoxic and hypoxic conditions. The process of adipogenic differentiation were divided into five timepoints including the stemness stage (D0), differentiating stage (D1 and D3), and specialized stage (D7 and D11). Cells in the different stages of adipogenesis were subjected to hypoxia for 24 h (1% O_2_) in advance, then some phenotypic characteristics were detected and cell sample were collected. Oxygen percentage under normoxic and hypoxic conditions is displayed in white (21% O_2_) and red boxes (1% O_2_), respectively. **(C)** Flow chart of cell phenotype detection and RNA-seq analysis.

*Cricetulus griseus*, commonly known as Chinese Hamster, has a typical tendency of spontaneous diabetes (Miedel and Hankenson, 2015; Wang et al., 2019). Many lipid-related metabolisms of the hamster are more similar to that of humans than mice or rats, including HFD-induced high plasma triglycerides, VLDL and apolipoprotein (ApoB) (Song et al., 2010). Meanwhile, hamster have similar lipid profiles to human, thus the hamster is considered to be an appropriate animal model for research of obesity-associated metabolic disorders, such as insulin resistance, hepatic steatosis, and dyslipidemia (Basciano et al., 2009; Zou et al., 2015; Sun et al., 2019). Considering that primary preadipocytes are most likely to recapitulate the hypoxic response during adipogenesis in a cell-autonomous fashion, we isolated preadipocytes from hamster inguinal WAT (iWAT) and established a preadipocyte differentiation model induced by the classic cocktail method. The differentially hypoxic response of preadipocytes during adipogenesis was evaluated and the expression of mRNAs and lncRNAs in the same Ribo-Zero RNA-seq libraries were analyzed to comprehensively elucidate the distinct effects of hypoxia during adipogenesis (Figure 1). This study will further uncover novel regulators potentially involved in WAT function and may provide a new viewpoint for interpretation and treatment of obesity.

## Results

### Hypoxic Response of Preadipocytes During Adipogenesis

**FIGURE 2:**
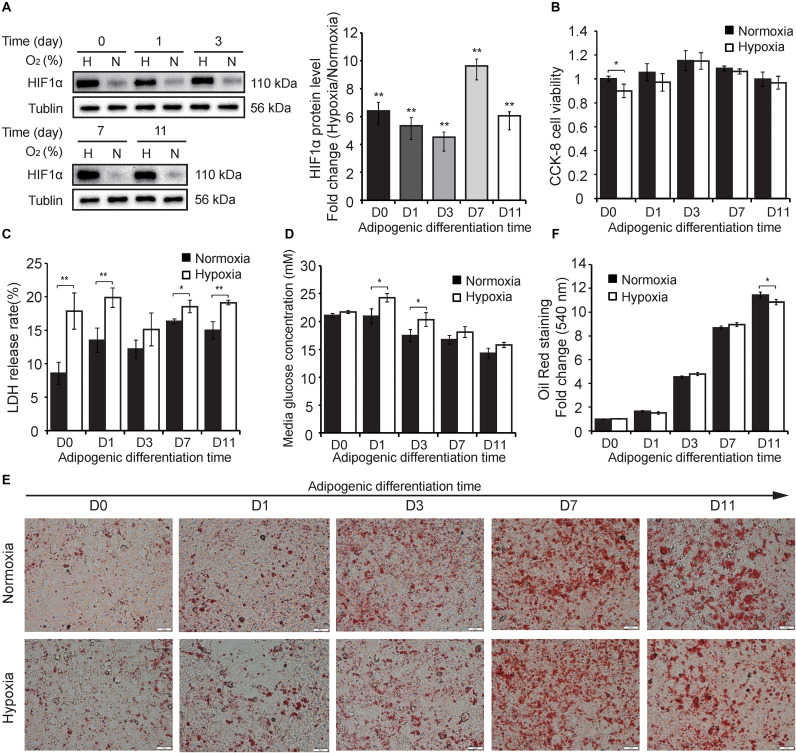
Hypoxic responses of cells during adipogenesis. **(A)** Western blot analyses of HIF-1α protein (left panel). Band intensities were quantified using ImageJ software (right panel) and Tubulin was used as a control. Cell viability **(B)**, membrane damage **(C)**, and the medium glucose concentration **(D)** were evaluated in cells at different stages of adipogenesis under hypoxia (1% O_2_) and normoxia (21% O_2_). **(E)** The cells at different stages of adipogenesis were stained by ORO and were photographed (scale bar: 20 μm). **(F)** Triglyceride in stained cells were quantified by measuring the absorbance at 510 nm. Three independent experiments were performed in triplicate. Means ± SD are presented for each group. *p < 0.05, **p < 0.01; hypoxia group compared with the normoxia-matched group at each timepoint. H, hypoxia; N, normoxia.

To explore the differently hypoxic responses of preadipocytes during adipogenesis, we divided this process into five timepoints including the stemness stage (D0), differentiating stage (D1 and D3), and specialized stage (D7 and D11) (Figure 1B). Cells in the different stages of adipogenesis were subjected to hypoxia for 24 h (1% O_2_) in advance, and then some phenotypic characteristics, including HIF-1α protein expression, cell viability, membrane integrity, medium glucose, and triglycerides, were evaluated. As shown in Figure 2, continuous hypoxic exposure for 24 h increased HIF-1α protein expression at all timepoints (*p* < 0.01; Figure 2A) and induced varying degrees of cell injury compared with normoxia-matched groups, including decreased cell viability mainly at D0 (*p* < 0.05; Figure 2B) and increased cell membrane damage at almost all stages of adipogenesis (Figure 2C). Furthermore, we found that hypoxia promoted glucose retention in the medium and inhibited cellular glucose absorption particularly in the early stages of adipogenesis (D1 and D3, *p* < 0.05; Figure 2D), this effect were consistent with reported mechanism that hypoxia affecting the expression and function of caveolins, decreasing cellular sensitivity to insulin, and then reducing effective glucose transport (Varela-Guruceaga et al., 2018; Wilson and Matschinsky, 2019). ORO staining showed that intracellular triglyceride accumulation in normoxia and hypoxia groups were increased, but hypoxia reduced triglyceride accumulation compared with the normoxia group at D11 (*p* < 0.05; Figures 2E,F), demonstrating that the primary cells isolated in this study were genuine preadipocytes and suggesting inhibitory effects of hypoxia on adipogenesis, which were consistent with a previous report (Varela-Guruceaga et al., 2018). These results indicate that hypoxic exposure for 24 h might lead to differently hypoxic responses of preadipocytes during adipogenesis.

### Hypoxia Influences Expression Profiles of mRNAs During Adipogenesis

**FIGURE 3:**
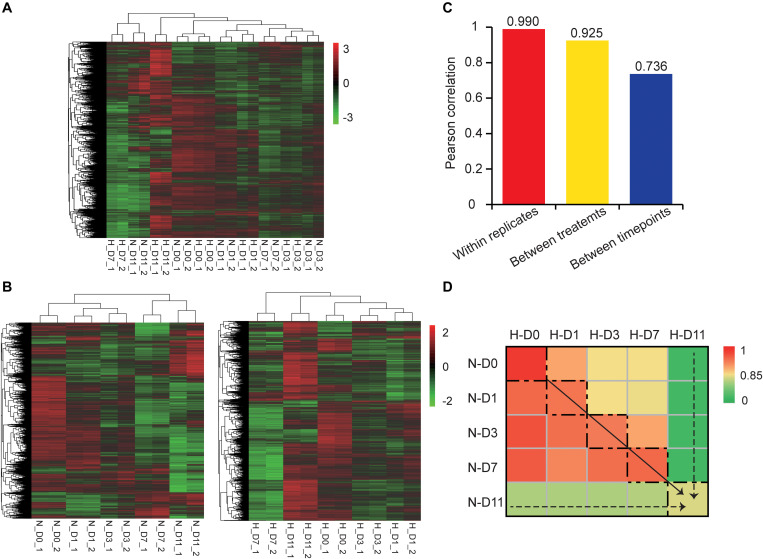
Hypoxia influences expression profiles of mRNAs during adipogenesis. Hierarchical clustering based on global expression profiles of mRNAs in all samples **(A)**, and hypoxia groups or normoxia groups **(B)**. **(C)** Pearson correlation coefficients within replicates of the same group, between two treatments, or between five timepoints of adipogenic differentiation. **(D)** Pearson correlation matrix between hypoxia and normoxia groups ranked by the adipogenic differentiation time. Dashed box represents the Pearson correlation between hypoxia and normoxia-matched groups. H, hypoxia; N, normoxia.

To explore changes in the mRNA transcriptome of preadipocytes during adipogenesis under hypoxia, we performed RNA-seq analysis of the hypoxia-exposed cells (normoxia-matched) at different stages of adipogenesis. From the twenty Ribo-zero RNA-seq libraries, we obtained ∼207.01 Gb (∼10.35 Gb per library) of high-quality data in which ∼196.21 Gb (∼9.81 Gb per library) were mapped to *Cricetulus griseus* reference genome with an average 94.78% map ratio (Supplementary Table S1). In total, 10,318 mRNAs were expressed (TPM > 1 in at least two replicates of one group) in twenty libraries. Hierarchical clustering based on global expression profiles of all samples showed that the two replicates of each group were clustered together and each timepoint, except D7 and D11, was clustered (Figure 3A). Hierarchical clustering in hypoxia and normoxia groups also suggested good biological replicates and that the early stages of adipogenesis (D0, D1, and D3) were clustered together and hypoxia obviously influenced the cluster profile of the late stages of adipogenesis (D7 and D11) (Figure 3B). Moreover, the average Pearson correlation coefficient of mRNAs showed that the effects of different stages during adipogenesis induced higher expression variability of mRNAs, followed by hypoxic exposure, and the biological replicates had a high correlation in the mRNAs expression profiles (Figure 3C). We also noted that hypoxia decreased the mRNA expression correlation as adipogenic differentiation was prolonged, particularly at the specialized stage (Figure 3D). Taken together, these results further demonstrate the success and repeatability of the adipogenesis model of hamster primary preadipocytes, and that hypoxia induced divergent changes of mRNA expression profiles during adipogenesis. Specifically, the higher the degree of cell differentiation, the greater the effect of hypoxia on the mRNA transcriptome.

### Functional Analysis of Differentially Expressed mRNAs (DE mRNAs)

**FIGURE 4:**
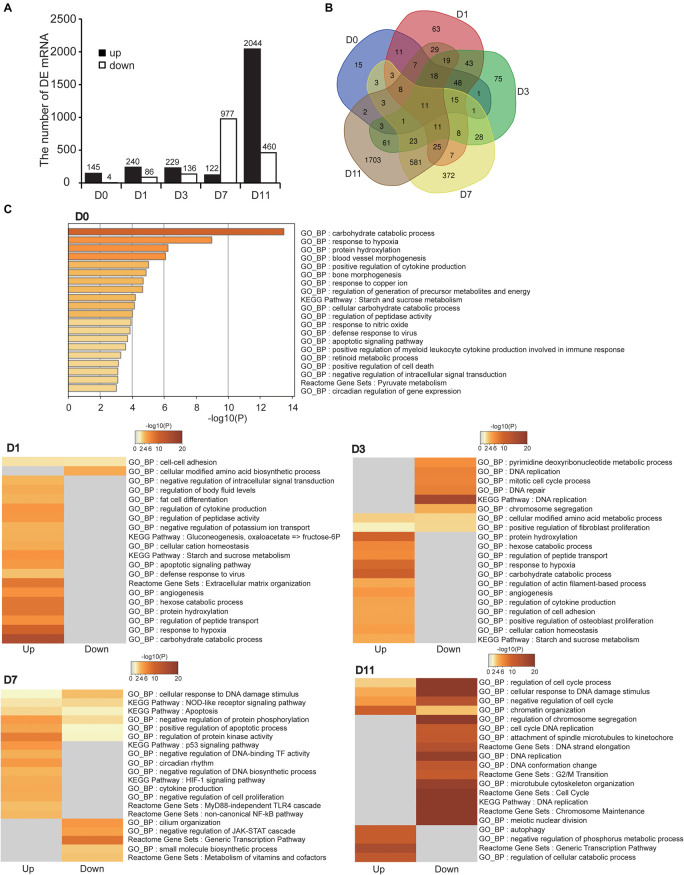
Identification and functional analysis of DE mRNAs. **(A)** Numbers of DE mRNAs at five timepoints of adipogenic differentiation. **(B)** Common and specific DE mRNAs in the five timepoints of adipogenesis showed by Venn diagram. **(C)** Functional enrichment analysis of DE mRNAs at the five timepoints of adipogenesis. Both upregulated and downregulated mRNAs were used in D1, D3, D7, and D11, and only upregulated mRNAs were used in D0.

We analyzed DE mRNAs at each timepoint (hypoxia *vs.* normoxia) with the cutoff (fold change > 2; FDR < 0.05). In total, 3,198 DE mRNAs were obtained at the five timepoints, 149, 326, 365, 1,099, and 2,504 DE mRNAs at D0, D1, D3, D7, and D11, respectively (Figure 4A and Supplementary Table S2). The number of DE mRNAs was gradually increased during cell differentiation, implying that hypoxia had a greater effect on more specialized cells, which was consistent with above results (Figure 3D). Figure 4B shows the overlapped and specific DE mRNAs in the different stages of adipogenesis. The overlapped DE mRNAs in the five timepoints were mainly related to basic cell activity and metabolism such as proteolysis (*Gapdh*, *Meltf*, *Gsn*, and *Aim2*), cell secretion (*Gapdh*, *Cxcl12*, and *Aim2*), small molecule biosynthesis (*Gapdh*, *Upp1*, and *Ptges*), and cell adhesion (*Cxcl12*, *Meltf*, and *Gsn*), suggesting that hypoxia affected basal cellular metabolism throughout adipogenesis.

The cellular adaption to hypoxia is mediated through a variety of mechanisms, mainly by increasing oxygen delivery (erythropoiesis, iron metabolism, and angiogenesis), reducing oxygen consumption (promoting anaerobic metabolism and inhibiting the TCA cycle), and regulating cell proliferation and apoptosis (Brahimi-Horn et al., 2007; Kaelin and Ratcliffe, 2008). Functional enrichment analysis of DE mRNAs at each timepoint revealed that these DE mRNAs were more or less related to hypoxia-adaptive categories (Figure 4C and Supplementary Table S3). For example, upregulated genes were directly enriched in response to hypoxia (or HIF-1 signaling pathway) at D0, D1, D3, and D7, angiogenesis (or blood vessel morphogenesis) at D0, D1, and D3, and energy metabolism at D0, D1, D3, D7, and D11. These well-known biological pathways of the hypoxic response may represent the shared mechanism of the cellular hypoxic response at different stages of adipogenesis. In addition, the cell differentiation process is usually accompanied by inhibition of cell proliferation and mild apoptosis (Bryja et al., 2004; Lamkanfi et al., 2006). We found that apoptosis-related pathways were enriched at D0, D1, and D7, and negative regulation of cell proliferation at D7, suggesting that further inhibition of proliferation and promotion of apoptosis were involved in the cellular response to hypoxia. It has been reported that autophagy, particularly mitophagy, is implicated in adipogenesis (Zhang Y. et al., 2019). We noted that the upregulated genes at D11 were related to autophagy, demonstrating that hypoxia aggravated autophagy in mature adipocytes (specialized stage). Intriguingly, immune-related pathways were enriched at D7, including cytokine production (inflammatory response), the MyD88-independent TLR4 cascade, and NF-κB pathway, implying that cells at the late stage of differentiation in response to hypoxia promoted the inflammatory response in WAT, which is also considered to induce WAT dysfunction (Crewe et al., 2017). It is noteworthy that DE mRNAs were enriched in the cell cycle, chromatin reorganization, DNA replication, and DNA repair at D11. These biological processes are involved in maintaining stemness of pluripotent cells. Hypoxia has been reported to help maintain cell stemness and regulate self-renewal partially by mediating the activity of stem cell factors (Oct4, c-Myc, and Nanog) (Li and Rich, 2010; Vadde et al., 2017). Therefore, we speculated that hypoxia may induce recovery of stemness in mature adipocytes (specialized stage). These results indicate that cells at the different stages of adipogenesis have shared and specific hypoxia adaptation mechanism.

### Hypoxia Differentially Modulates the Expression Profiles of Adipose-Associated Genes

**FIGURE 5:**
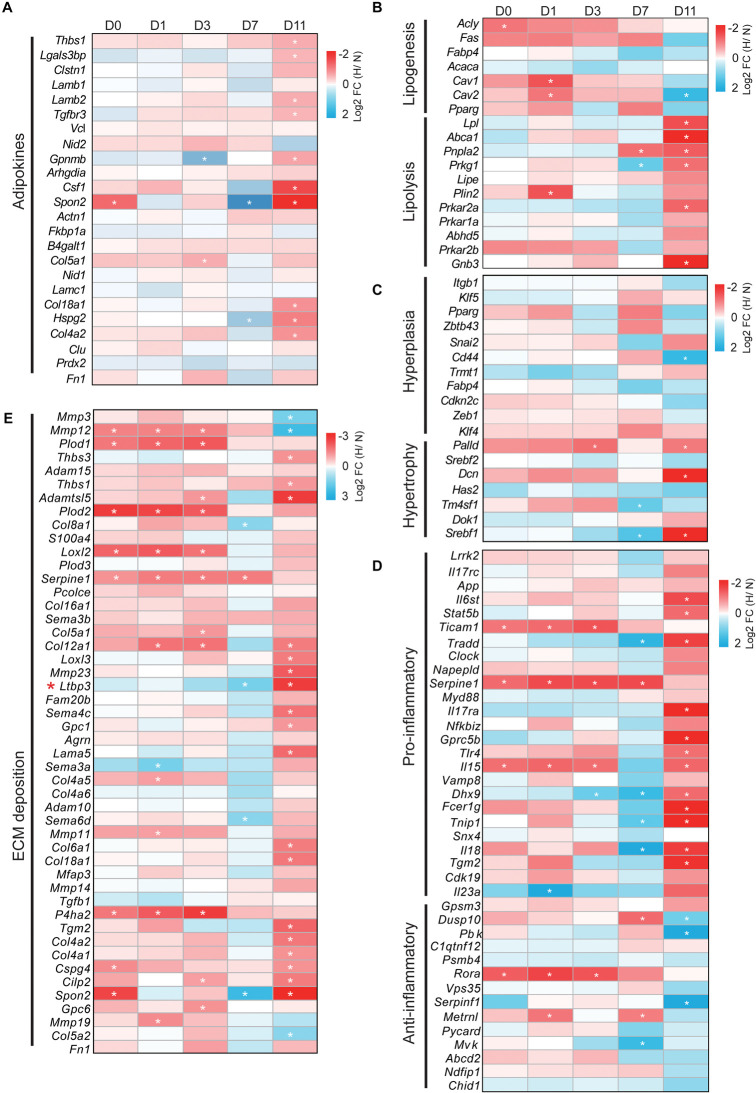
Hypoxia differentially modulates the expression profiles of adipose-associated genes. Genes associated with **(A)** adipokines, **(B)** lipogenesis and lipolysis, **(C)** hyperplasia and hypertrophy, **(D)** pro-inflammation and anti-inflammation, and **(E)** ECM modulated by hypoxia. Expression profiling of these genes is presented as heatmaps [log_2_ fold change (hypoxia/normoxia)]. Corresponding box plots represent the overall expression change of these genes at each timepoint (Supplementary Figure S1). *p < 0.05, **p < 0.01. H, hypoxia; N, normoxia. The gene (*Ltbp3*) marked by red star mentioned in “Discussion” section.

We next determined whether adipose-associated genes involved in adipokines, lipogenesis, lipolysis, hyperplasia, hypertrophy, inflammation, and ECM were altered by hypoxia. The expression profile changes of these genes (only one-to-one hamster ortholog genes were used) were represented as a heatmap [log_2_ fold change (hypoxia/normoxia)]. WAT regulates the whole body metabolism by secreting adipokines involved in ECM organization, cell adhesion, and the inflammatory response (Dahlman et al., 2012). We found that hypoxia increased the expression of most adipokines during cell differentiation except at D7. In particular, hypoxia significantly upregulated these adipokines at D11 compared with the other timepoints (*p* < 0.01), implying that mature lipid-filled adipocytes had a stronger secretion capacity under hypoxia (Figure 5A, Supplementary Figure S1A and Supplementary Table S4-1). Interestingly, the expression of most adipokines was decreased at D7 (*p* = 0.171, 0.055, 0.065, and 0.004 compared with D0, D1, D3, and D11, respectively). This suggests that the lipid-unfilled cells with a mature adipocyte identity had a divergent expression pattern of adipokines compared with mature lipid-filled adipocytes and immature adipocytes at different adipogenesis stages under hypoxia.

Next, we analyzed how hypoxia affected the expression of adipose metabolism-related genes, including lipogenesis, lipolysis, hyperplasia, and hypertrophy, in cells at the different adipogenesis stages. As shown in Figure 5B, Supplementary Figure S1B, and Supplementary Table S4-2, hypoxia increased the expression of lipogenesis genes at D0, D1, D3, and D7, and markedly decreased their expression at D11. In contrast, the expression of lipolysis genes was weakly altered at D0, D1, and D3, but were significantly downregulated at D7 and upregulated at D11. This result shows that mature lipid-filled adipocytes had a strong lipolysis capacity and the lipogenesis capacity increased in cells at other adipogenesis stages under hypoxia. In parallel, we found that hypoxia weakly influenced the expression pattern of hyperplasia- and hypertrophy-related genes at D0, D1, and D3 (Figure 5C, Supplementary Figure S1C, and Supplementary Table S4-3). However, hypoxia conversely altered the expression of these genes at D7 and D11, although the overall difference was not significant. Specifically, hypoxia increased hyperplasia-related gene expression at D7 and decreased such expression at D11, and hypertrophy-related genes showed the opposite trend (Figure 5C and Supplementary Figure S1C). These results indicate that hypoxia had little effect on the adipose metabolism process in the early stage of adipogenesis and intensively influenced adipose metabolism in mature adipocytes, further suggesting that hypoxia had the opposite effects in mature lipid-filled adipocytes and lipid-unfilled adipocytes.

An appropriate inflammatory response and ECM deposition are important characteristics of WAT homeostasis (Crewe et al., 2017; Hammarstedt et al., 2018). We also explored how hypoxia affected the expression of inflammation- and ECM-related genes in cells at different adipogenesis stage. Hypoxia had a weak and similar effect on the inflammatory response (pro-inflammation and anti-inflammation) at D0, D1, and D3 (Figure 5D, Supplementary Figure S1D and Supplementary Table S4-4), and significantly increased the expression of pro-inflammatory genes and decreased that of anti-inflammatory genes at D11. Intriguingly, it appeared that hypoxia had the opposite effect on the inflammatory response at D7 compared with D11. Specifically, pro-inflammatory gene expression was markedly decreased and anti-inflammatory gene expression showed an increased trend. In addition, hypoxia obviously increased the expression of ECM deposition-related genes at D0, D1, D3, and D11, but it was decreased at D7 (Figure 5E, Supplementary Figure S1E and Supplementary Table S4-5). These data indicate that hypoxia induced intense changes of inflammation and ECM in mature lipid-filled adipocytes, and lipid-unfilled adipocyte had the opposite hypoxic response compared with cells at other adipogenesis stages.

### Characteristics and Expression Profiles of lncRNAs

**FIGURE 6:**
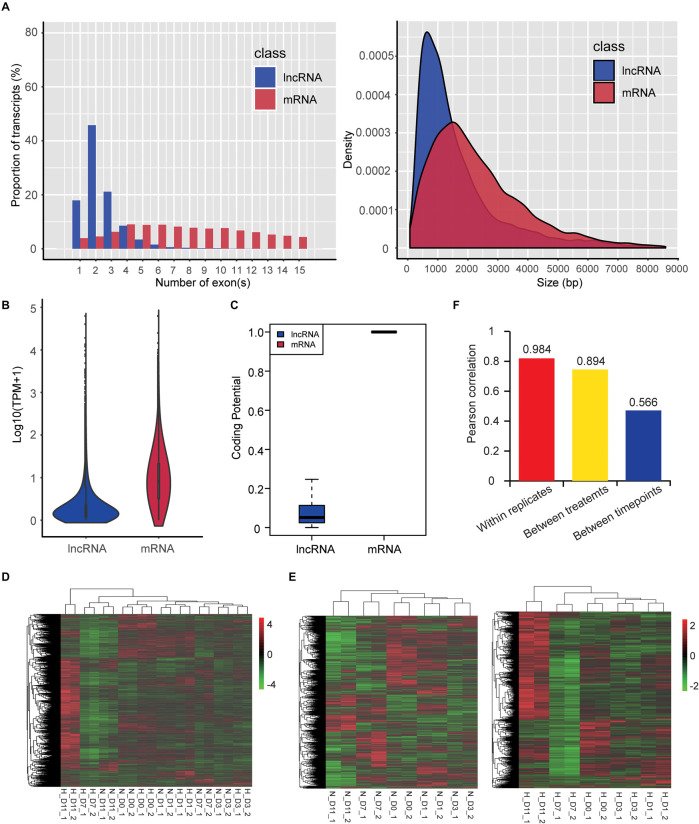
Characteristics and expression profiles of lncRNAs. **(A)** Exon number (transcripts with 1–15 exons were included) and length of transcripts (bp: base pair), **(B)** expression level [TPM value transformed by log_10_ (TPM + 1)], and **(C)** coding potential. Hierarchical clustering based on global expression profiles of lncRNAs in all samples **(D)**, and hypoxia or normoxia groups **(E)**. Pearson correlation coefficients within replicates of the same group, between two treatments, or between five timepoints of adipogenic differentiation **(F)**.

We also identified 4,296 lncRNAs that were substantially expressed (TPM > 0.1 in at least two replicates of one group). Characteristic comparison of mRNAs and lncRNAs showed that lncRNAs had more transcripts with a lower exon number (median value = 2) and shorter transcript length (median value = 1,200 nt) (Figure 6A), lower expression level (mean TPM value = 4.68; Figure 6B), and lower coding potential (Figure 6C) compared with mRNAs (median exon number = 10; median transcript length = 2,012 nt; mean TPM value = 20.44), which were consistent with a previous study (Jin et al., 2018). Hierarchical clustering based on global expression profiles of lncRNAs indicated good biological replicates in each group (Figure 6D), and that the early stages of adipogenesis (D0, D1, and D3) were clustered together (Figure 6E) and hypoxia obviously influenced the expression profile of lncRNAs in the late stages of adipogenesis (D7 and D11) (Figure 6E). Moreover, the average Pearson correlation coefficient of lncRNAs showed that the effects of the different stages during adipogenesis induced higher expression variability of lncRNAs, followed by hypoxic exposure, and the biological replicates had a high correlation (Figure 6F). This tendency was consistent with the mRNA expression profiles (Figure 3C). These results systemically identified the lncRNA transcriptome in hamster preadipocytes during adipogenesis.

### Identification and Functional Analysis of DE lncRNAs During Adipogenesis Under Hypoxia

**FIGURE 7:**
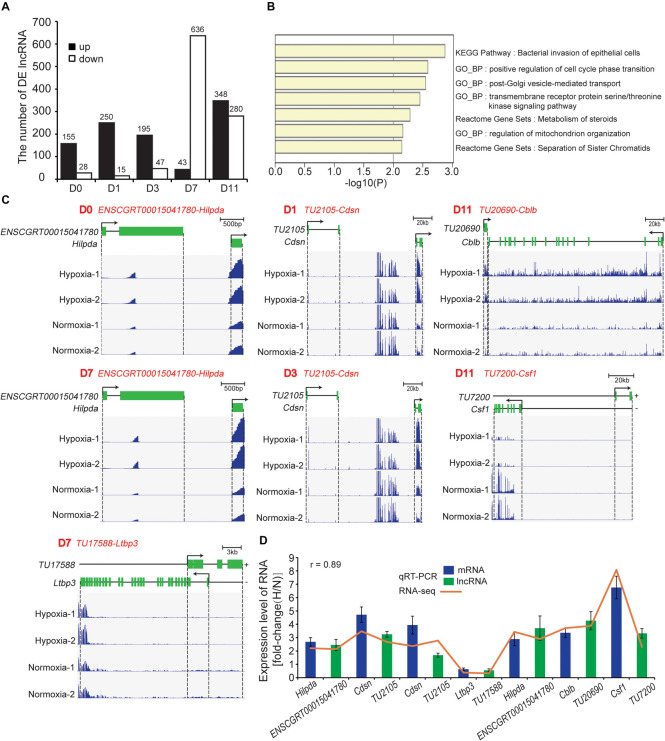
Identification and functional analysis of DE mRNAs. **(A)** Number of DE lncRNAs at the five timepoints of adipogenesis. **(B)** Functional enrichment analysis of combined DE mRNAs that were highly correlated and simultaneously locus-nearby with DE lncRNAs. **(C)** Loci and expression levels of lncRNA and mRNA. *ENSCGRT00015041780*-*Hilpda* (D0 and D7), *TU2105*-*Cdsn* (D1 and D3), *TU17588*-*Ltbp3* (D7), *TU20690*-*Cblb* (D11), and *TU7200*-*Csf1* (D11), respectively. **(D)** Seven lncRNA-mRNA pairs were selected to validate RNA-seq results using qRT-PCR. H, hypoxia; N, normoxia.

We identified 1,431 DE lncRNAs at all timepoint (hypoxia *vs.* normoxia) with the cutoff (fold change > 2; FDR < 0.01). Among them, 158, 250, 195, 43, and 348 lncRNAs were upregulated and 28, 15, 47, 636, and 280 lncRNAs were downregulated at D0, D1, D3, D7, and D11, respectively (Figure 7A and Supplementary Table S5). To predict the biological function of DE lncRNAs, *tans*/*cis* analysis was performed to identify highly correlated (|r| > 0.9, *P* < 0.05; Supplementary Table S6) and locus-nearby mRNAs (within 100 kb of DE lncRNAs; Supplementary Table S6). We found that 2, 3, 5, 13, and 50 DE mRNAs were highly correlated and simultaneously locus-nearby with DE lncRNAs at D0, D1, D3, D7, and D11, respectively (Supplementary Table S6). Functional enrichment analysis of these combined mRNAs suggested that they were mainly involved in the cell cycle, vesicle-mediated transport, and mitochondrion organization (Figure 7B). Previous studies have demonstrated that hypoxia participates in regulation of the cell cycle and induction of cell stemness (Mohyeldin et al., 2010), and mediates extracellular vesicle-loaded adipokine secretion (Mleczko et al., 2018), triggering mitochondrial dysfunction in adipocytes (Jang et al., 2013). Such findings imply that these DE lncRNAs are involved in the regulation of adipocyte functions under hypoxia.

We next identified 28 one-to-one lncRNA-mRNA pairs that were less than 100 kb in distance and positively correlated with the expression level based on the above results of *tans*/*cis* analysis (Supplementary Table S6). Notably, some mRNAs have been reported to be closely related to adipocyte functions. For example, hypoxia-inducible lipid droplet-associated (Hilpda), also referred to as HIG-2, localizes to lipid droplets in adipocytes and is the common target gene of HIF-1 (Gimm et al., 2010) and PPARγ (Montserrat and Kersten, 2017). Hilpda promotes lipid deposition in adipose tissue and improves diet-induced insulin resistance independent of lipolysis (DiStefano et al., 2016). We found that the expression levels of lncRNA *ENSCGRT00015041780* and *Hilpda* were increased significantly at D0 and D7 under hypoxia (Figure 7C), indicating that *ENSCGRT00015041780* might regulate *Hilpda* expression. A previous study has indicated that hypoxia induces recruitment of brite adipocytes and “browning” of WAT by stimulating lactate production (Trayhurn and Alomar, 2015). Interestingly, lncRNA *TU2105* and *Cdsn*, a brite adipocyte marker (Wang et al., 2016), were consistently upregulated at D1 and D3 under hypoxia (Figure 7C), suggesting that *TU2105* is involved in the regulation of hypoxia-induced browning of white adipocytes. In addition, Ltbp3 interacts with fibrillin-1 and is closely associated with ECM deposition (Robertson et al., 2015). The *TU17588-Ltbp3* pair was downregulated at D7 under hypoxia (Figure 7C), indicating that *TU17588* may be related to WAT fibrosis and lipid-unfilled cells with a mature adipocyte identity have a “healthier” response to hypoxia (Figure 5E: marked by red star). It is noteworthy that *TU20690-Cblb* and *TU7200-Csf1* were simultaneously upregulated at D11 under hypoxia (Figure 7C). Csf1 and Cblb are reported to be involved in the infiltration and activation of macrophages (Hirasaka et al., 2007; Abe et al., 2013). Therefore, these identified lncRNA-mRNA pairs may represent a crucial regulation axis in the cellular response to hypoxia during adipogenesis. Next, we validated the expression levels of the abovementioned pairs by qRT-PCR, which showed a high correlation between RNA-seq and qRT-PCR results (Figure 7D).

## Discussion

In recent years, hypoxia has been widely reported to occur in the WAT local microenvironment during obesity (Trayhurn, 2013). Adipocytes have a life cycle that reflects the different stages of adipogenesis in the WAT niche (Jiang et al., 2012; Lynes and Tseng, 2018). Some studies have indicated that preadipocytes and mature adipocytes have different hypoxic responses, but did not further extend to deeper experiments to the entire period of adipogenesis (Wang et al., 2008; Rosenow et al., 2013). In this study, hamster primary preadipocytes were isolated and induced to undergo adipogenic differentiation. Although there was no obvious alteration in adipogenic differentiation or triglyceride deposition except at D11, the cells in the five stages of adipogenesis presented specific hypoxic responses, which prompted us to explore the underlying mechanisms at the transcriptome level by RNA-seq. Whether at the mRNA or lncRNA transcriptome level, Pearson correlation at the different stages of adipogenesis was lowest and the hypoxic exposure have lower expression correlation compared with the biological replicates, reflecting the reliability of the experiment and the effectiveness of the hypoxic treatment (Figures 3C, 6F). Moreover, the numbers of DE mRNAs and DE lncRNAs were gradually increased as the cells continued to differentiate (Figures 4A, 7A), implying that hypoxia has a greater effect on more specialized adipocytes. These results have prompted us to consider the effect of more detailed cell stages during differentiation in a future study.

Obesity-induced local hypoxia in WAT has been demonstrated to be involved in insulin resistance, inflammation, and fibrosis. However, the contribution of cells at different stages of adipogenesis to these biological processes remain to be elucidated. This study showed that hypoxia induced HIF-1α protein expression in all cells at different stages of adipogenesis (Figure 2A), and subsequently triggered varying degrees of hypoxia response during adipogenic differentiation, including cell viability, LDH release, glucose absorption (Figures 1B–D). These results enticed us to explore the underlying mechanisms of differentially hypoxic response of preadipocytes during adipogenesis and its effect on the expression of adipose-related genes at the molecular level. We found that DE mRNAs at the five timepoints of adipogenesis were more or less directly enriched in hypoxia-associated pathways (Figure 4C), but adipose-related pathways were differentially enriched, which implied that the cells at the different stages of adipogenesis could be response to hypoxia, but had different effects on adipose-related identity. When responding to excessive nutrients and energies, WAT expanded in one of two ways: hyperplasia of resident precursors to new adipocytes and hypertrophy of existing adipocytes. Hyperplastic expansion is generally considered as metabolism-adaptive and healthy, whereas hypertrophic expansion is metabolically unhealthy and accompanied by an increase of the hypoxic degree, which further exacerbate local insulin resistance, inflammation, and fibrosis (Ghaben and Scherer, 2019). In our study, the greater variation of adipose-associated genes occurred in cells at late stages of adipogenesis, which was consistent with the results that hypoxia had a greater effect on more specialized adipocytes (Figure 3D). Interestingly, we found that lipid-unfilled cells with a mature adipocyte identity had a divergent expression pattern of all analyzed gene sets compared with the other stages of adipogenesis after hypoxic exposure. Previous studies have indicated that new adipocytes formed via hyperplasia of resident precursor are mature and highly plastic adipocytes with a great potential for lipid storage (Crewe et al., 2017; Hammarstedt et al., 2018). Thus, based on previous reports and the results of the adipose-related gene set analysis in this study, we tentatively speculated that the newly matured and lipid-unfilled adipocyte (corresponding to the D7 of differentiation) have a high plasticity and ability to maintain homeostasis, and these cells could be able to alleviate hypoxia-induced inflammation, fibrosis, insulin resistance, and exhausted lipid storage in WAT. In contrast, the cells at the late stages of adipogenesis (corresponding to the D11 of differentiation) were mature and hypertrophic adipocytes that exhibiting an exhausted lipid storage potential, an aggravated inflammatory response and insulin resistance. Thus, these hypertrophic adipocytes have unhealthy effects on adipose functions under hypoxia (Ghaben and Scherer, 2019). In addition, cells at the early stages of adipogenesis are not genuine adipocytes and have no or an insufficient capacity to store fat, and thus show little unhealthy effects. These results support the previous view that hyperplasia is generally metabolism-healthy, and hypertrophy is metabolically unhealthy in adipose tissue. Therefore, this study suggests that increasing the proportion of newly matured and lipid-unfilled adipocytes with strong plasticity may be an effective strategy for the treatment of obesity-related disorders.

LncRNAs, a class of non-coding transcripts longer than 200 nt, are emerging as key regulators involved in a wide variety of biological processes (Shih and Kung, 2017). In the present study, we analyzed the expression pattern of mRNAs and lncRNAs in the same library and further explored the relationships of lncRNAs and mRNAs in terms of the genomic location and expression level. We identified 28 lncRNA-mRNA pairs that might be involved in the cellular response to hypoxia at different stages of adipogenesis. Among them, *Hilpda* (DiStefano et al., 2016), *Cdsn* (Wang et al., 2016), *Ltbp3* (Robertson et al., 2015), *Cblb*, and *Csf1* (Hirasaka et al., 2007; Abe et al., 2013) have been reported to be involved in adipose functions. These lncRNA-mRNA pairs represent the crucial regulation axis in the cellular response to hypoxia during adipogenesis. Vamp2 regulates glucose uptake by activating the Akt-Vamp2-Glut4 pathway (Guo et al., 2019). Many members of the Fox family have been implicated as important regulators of metabolism. Foxf2 regulates glucose homeostasis through lowering the levels of IRS1 and decreasing insulin-mediated glucose uptake in adipose tissue (Westergren et al., 2009). In addition, Tmem150a plays a key role in the regulation of cytokine production downstream of TLR4 (Romanet, 2017) and may be involved in the inflammatory response in adipose tissue. Firstly, lipid-related metabolisms of the hamster are more similar to that of humans than mice or rats, hamster have similar lipid profiles to human, the hamster is recently considered to be an appropriate animal model for research of obesity-associated metabolic disorders. Secondly, it is worth noting that of 28 lncRNA-mRNA pairs, 17 lncRNAs have sequence similarity to humans greater than 80%. The high sequence similarity of lncRNA is the basis of highly similar functions (Johnsson et al., 2014). Thus, we deduced that these lncRNA-mRNA pairs identified in the present study might be crucial regulators responsible for the differentially hypoxic response of cells during adipogenesis, and have important guiding significance for the interpretation and intervention of human obesity and its related diseases. Deep functional studies are needed to further validate these regulatory pathways.

However, there are some limitations in this study that can be improved in future research. First, the effects of hypoxia during adipogenesis were mainly elucidated by cell-based experiments *in vitro*, dissection of differentially hypoxic effects in obese animal models should be performed in the future. Moreover, the occurrence of obesity-induced hypoxia *in vivo* varies and is unpredictable, and the divergent effects of different hypoxia exposure times and different oxygen concentrations should be considered. In addition, adipose tissue has high cellular heterogeneity (Vijay et al., 2019) and apart from cells at the different stages of adipogenesis, the hypoxic response of other cell types such as macrophages (Catrysse and van Loo, 2018) or the crosstalk mechanism of different cell types and different cell stages after hypoxic exposure remain to be further elucidated.

## Conclusion

Taken together, this study dissected the effects of hypoxia on cells at different stages of adipogenesis, and found that hypoxia caused divergent changes of cell phenotypes during adipogenesis and differentially modulated the expression profile of adipose-associated genes. We also identified DE mRNAs and DE lncRNAs in differentially hypoxic responses of preadipocytes during adipogenesis. Notably, our study revealed some important lncRNA-mRNA regulation axis in the cellular response to hypoxia during adipogenesis. These results uncovered novel regulators that are potentially involved in WAT function and may provide a new viewpoint for interpretation and treatment of obesity.

## Materials and Methods

### Hamster Primary Preadipocyte Culture

Healthy female 6-week-old hamsters weighing 80 ± 10 g were bought from Dashuo Laboratory Animal Center (Chengdu, Sichuan, China) and housed in a standard environment (20 ± 2°C and 58% ± 2% humidity), with free choice feeding for three days before experiment. Primary preadipocyte culture was performed as previously described (Saulnier-Blache et al., 1991; Klaus et al., 2001). Briefly, hamsters were sacrificed by cervical dislocation (*n* = 15). iWATs were removed under sterile condition and the connective tissue and blood vessels were dissected out. The tissue from all hamsters were pooled and washed with PBS containing 4% penicillin-streptomycin (Sigma-Aldrich, Milwaukee, United States), cut into small pieces, and digested at 37°C water baths in DMEM supplemented with 1 mg/mL collagenase I (Sigma). The suspension was filtered through 70 and 40 μm strainer. Floating adipocyte fraction was discarded and the remaining preadipocyte fraction were collected by low speed centrifugation. Preadipocytes were seeded onto culture dishes and incubated in complete medium (CM) containing DMEM (Hyclone, Logan, UT, United States) and 10% FBS (GIBCO, Grand Island, NY, United States) at 37°C in a humidified atmosphere with 5% CO_2_ and 95% air until confluence (Figure 1A).

All animal experiments was conducted according to the Regulations for the Administration of A airs Concerning Experimental Animals (Ministry of Science and Technology, China, revised in June 2004) and was approved by the Institutional Animal Care and Use Committee at the College of Animal Science and Technology, Sichuan Agricultural University, Sichuan, China, under permit “No. DKY-B20171903.”

### Preadipocyte Differentiation and Hypoxia Exposure

Differentiation of preadipocytes was performed by classic cocktail methods (Zhang et al., 2018). Cells grown to 100% confluence (D0) were induced using differentiation medium (DM) containing CM, 1 M dexamethasone, 10 g/mL insulin and 0.5 mM IBMX (Sigma). Two days after the induction, medium was replaced to the maintenance medium (MM) containing CM and 10 g/mL insulin (Sigma). For hypoxia exposure, cells at five differentiation timepoints (D-1, D0, D2, D6, and D10) received hypoxia for 24 h in a modular incubator chamber with 5% CO_2_, 1% O_2_, and 95% N_2_ (MIC-101, Billups-Rothenberg, Del Mar, CA, United States). Corresponding medium in different groups were pre-equilibrated to hypoxia for at least 4 h before use. Cells in the normoxia-matched group were placed in conventional conditions (5% CO_2_ and 95% air) and served as the control (Figure 1B).

### Detection of Cell Viability and LDH Release

Cell viability and LDH release were respectively analyzed using CCK8 and LDH Cytotoxicity Assay Kit (Beyotime, Shanghai, China) according to the manufacturer’s recommendations. For CCK8 detection, 10 μL CCK8 reagent was added to the culture medium 4 h before analysis and then optical density (OD)450_nm_ values were measured using a microplate reader (Thermo Fisher Scientific, Madrid, Spain). For LDH release analysis, the culture medium in each group was premixed with the relevant reagent and incubated. OD490_nm_ values were measured and LDH release rate presented as the percentage of the maximum enzymatic activity. At least three independent experiments were repeated three times. All values are presented as mean ± standard deviation (SD).

### Detection of Culture Medium Glucose

Culture medium glucose content were detected using a glucose oxidase–peroxidase assay kit (Nanjing Jiancheng Bioengineering Institute, China). Briefly, cells in different timepoints were gently washed twice with prewarming (37°C) PBS and treated with insulin (100 nM) in complete medium for 24 h. Culture medium of 3μL each group were premixed with 300 μL work solution and incubated for 15 min. OD 505_nm_ values were measured using a microplate reader. Blank control and adjustment group added to 3 μL sterile distilled water and 3 μL calibrator (5.55 mM), respectively. The content of culture medium glucose in normoxia group was used as a reference to determine the hypoxia effect. At least three independent experiments were repeated three times. All values are presented as mean ± SD.

### Oil Red O Staining and Triglyceride Assay

Briefly, the cells in different timepoints were gently washed with PBS, fixed with 4% paraformaldehyde for 60 min, and then washed with PBS for three times. The fixed cells were stained with 0.5% ORO for 3 h at room temperature and washed with PBS for three more times. Subsequently, images were captured using an Olympus IX53 microscope (Olympus, Tokyo, Japan). For the triglyceride assay, the stained cells were incubated with isopropanol for 20 min, and OD values were measured at a wavelength of 510_nm_ by a spectrophotometer.

### Total RNA Extraction, Library Preparation, and Sequencing

Total RNA was isolated from the cell sample using HiPure Total RNA Mini Kit (Magen, Guangzhou, China) according to the manufacturer’s instruction. The integrity and quality of total RNA samples were analyzed with NanoDrop 2000 (Thermo Fisher Scientific, Wilmington, DE, United States) and Bioanalyzer 2100 system (Agilent Technologies, Palo Alto, CA, United States). The RNAs with a ratio of absorbance at 260/280 nm ranged from 1.8 to 2.0 and RIN value > 1.8 were selected for further study. A total of twenty samples (hypoxia vs. normoxia, five timepoints, two replicates) were selected for sequencing. Approximately 1 μg total RNA were used to generate RNA-seq libraries using the Ribo-Zero^TM^ kit (Epicenter, Madison, WI, United States) for each sample, and then high-quality strand-specific libraries were sequenced on the MGI-SEQ 2000 platform (BGI, Shenzhen, Guangdong, China) and 100-bp paired-end reads were obtained. High-quality data were controlled by removing poly-N and low-quality reads including those with ≥ 10% unidentified nucleotides, > 10 nt aligned to the adapter with ≤ 10% mismatches allowed, and with > 50% of bases with phred quality < 5. The RNA-Seq data have been deposited in NCBI’s (National Center for Biotechnology Information) Gene Expression Omnibus (GEO) and the accession number was GSE143873.

### Identification of mRNA and lncRNA

Clean data were mapped to the *Cricetulus griseus* reference genome (CriGri-PICR) using STAR (v.2.6.0c) with suggested parameters in GENCODE to identify mRNA transcripts (Ghosh and Chan, 2016). To obtain lncRNAs transcripts, the mapped reads for each sample were assembled using Cufflinks (v.2.1.1) (Pertea et al., 2015). Then we performed a transcript’s filtering and merging with software AssemblyLine^1^ and TACO (v.0.7.3). First, transcripts with clipped exon (first or last exons < 15 bp) or poorly assembled (transcript length ≤ 250 bp) were removed. Second, remaining transcripts were filtered from background noise, then subjected to taco_run (main function in TACO) for transcript’s merging. The transcripts annotated as PCG (protein coding genes) were discarded and remaining (putative non-coding) transcripts were used for the prediction of coding potential according flowed steps: (1) putative non-coding transcripts sequences were translated in all six possible frames with Transeq (part of EMBOSS v.6.6.0) and count the significant domain hits against Pfam (release31) database with PfamScan (v.1.6) as well as coding sequence in reference genome. (2) Shared domains which hit by putative non-coding and coding sequence were considered to be likely with *p* > 0.05 or odd < 10 after fisher exact test, putatively non-coding transcripts that hit in likely domains were furtherly removed. (3) CPC2^2^ was used to assess coding potential in both strands of the putatively non-coding sequences (Kong et al., 2007). (4) Putative transcripts supported to be non-coding by PfamScan and CPC2 were considered to be lncRNAs.

### Expression Analysis of mRNA and lncRNA

The mRNA and lncRNA expression level of transcripts per kilobase million (TPM) of each sample was calculated by kallisto (v0.44.0) (Pertea et al., 2015); mRNAs with TPM > 1 in at least two replicates of one group were considered to be expressed (lncRNAs with TPM > 0.1). Then edge R (Bioconductor version: Release 3.10) (Robinson et al., 2010) was applied to detect DE mRNAs and lncRNAs, and mRNAs with adjusted-*P* values < 0.05 and |log_2_(FC)| > 1 were considered to be DE mRNAs. Given the large variation and low abundance in expression level, lncRNAs with adjusted-*P* values < 0.01 and |log_2_(FC)| > 1 were considered to be DE lncRNAs. Loci and expression levels of lncRNA and mRNA reads abundance were generated by integrative genomics viewer (IGV) v.2.4.10 (Thorvaldsdottir et al., 2013).

### Functional Enrichment Analysis

Gene Ontology (GO) functional enrichment analysis and KEGG pathway functional enrichment analysis were performed with DE mRNAs at the Metascape web portal^3^ (Zhou et al., 2019). To predict the functions of DE lncRNAs, the DE mRNAs that were within 100 kb of the lncRNAs were collected (*cis* analysis). Then, Hmisc (an R package)^4^ was applied to calculate Pearson correlations between lncRNAs and mRNAs, and mRNAs with high correlations (|r| > 0.90 and *p* < 0.05) were collected (*trans* analysis). We extracted the overlapping of highly related mRNAs from *cis*/*trans* analysis and performed functional enrichment analysis as well. GO terms or KEGG pathways with Benjamini-corrected *P* < 0.05 were significant.

### Gene Sets Analysis

Adipose-associated gene sets were quoted from previous reports, including adipokines (Dahlman et al., 2012), lipogenesis, lipolysis (Arner et al., 2018), hyperplasia, hypertrophy (Hammarstedt et al., 2018), inflammatory (Reilly and Saltiel, 2017) and ECM deposition (Naba et al., 2016). These genes were usually derived from human- or mouse-based studies and the genes which only one-to-one ortholog to hamster were used. Then the expression profile changes of these genes were presented by heatmaps [log_2_ fold change (hypoxia/normoxia)].

### Validation of mRNAs and lncRNAs by qPT-PCR

The reverse transcription of mRNA and lncRNA from total RNA were performed using PrimeScriptTM RT Reagent Kit with gDNA Eraser (Takara, Beijing, China). qPCR reaction system (in 10 μL) included 1 μL cDNA, 0.5 μL primers, 5 μL SYBR (TaKaRa, Shanghai, China), and 3 μL RNA-free water. The following RT-PCR reaction was performed in a Bio-Rad CFX96 PCR System (Bio-Rad, Hercules, United States) for all mRNAs and lncRNAs: 95°C for 3 min, followed by 40 cycles of 95°C for 10 s and amplification at the optimal temperature for each gene for 30 s, 95°C for 30 s, and then a melting curve analysis (65–95°C). The relative expression of mRNA and lncRNA was calculated using the 2^–ΔΔCT^ method and expressed as fold-change relative to the normoxia group. β*-actin* was used to correct the gene expression. All primers used for qPCR were listed in Supplementary Table S7.

### Western Blot Analysis

Western blot analysis was performed as previously described (Zhang J. et al., 2019). Total protein was extracted from the hypoxic and normoxic cells in different timepoints using radioimmunoprecipitation assay lysis buffer containing protease and phosphatase inhibitors (Beyotime, Beijing, China) and quantified using a BCA protein assay. Approximately 30 g of protein was loaded and separated on an 8% SDS-PAGE gel, and then transferred to polyvinylidene difluoride membranes (BIO-RAD, Hercules, United States). The membranes were blocked with non-fat milk for 2 h at room temperature, and then incubated with anti-HIF-1α (Abcam, Cambridge, United States) primary antibodies at 4°C overnight (dilution ratios 1:1000). Subsequently, the membranes were washed in PBS with Tween-20 before incubating with secondary antibodies for 2 h at room temperature. The antigen–antibody bands were visualized and quantified using ImageJ software (Bethesda, MA, United States). Tubulin (Abcam, Cambridge, United States) (dilution ratios 1:1000) was used as a control.

### Statistical Analysis

All experiments were performed as at least three independent experiments with three technical repetitions. The data are expressed as mean ± SD. Significance tests were performed using SPSS 22.0 software (SPSS, Chicago, United States). Unpaired Student’s *t*-test and one-way ANOVA with Tukey’s *post hoc* test was used to evaluate the differences between two groups or three or more groups, respectively. *p* < 0.05 was considered as statistically significant (^∗^*p* < 0.05, ^∗∗^*p* < 0.01).

## Data Availability Statement

The datasets presented in this study can be found in online repositories. The names of the repository/repositories and accession number(s) can be found in the article/ Supplementary Material.

## Ethics Statement

The animal study was reviewed and approved by the Institutional Animal Care and Use Committee at the College of Animal Science and Technology, Sichuan Agricultural University, Sichuan, China, under permit “No. DKY-B20171903.”

## Author Contributions

JZ, JM, and ML designed the experiments. JZ, JM, XZ, SH, JS, KL, LJ, and LL carried out the experiments data organization and statistical analyses. PL provided the experimental samples. JZ and JM wrote the original manuscript. SH, LJ and QT participated in the study design and discussed the manuscript. LG, XL, SS, and ML reviewed and edited the manuscript. All authors read and approved the final manuscript.

## Conflict of Interest

The authors declare that the research was conducted in the absence of any commercial or financial relationships that could be construed as a potential conflict of interest.

## Footnotes

1 http://assemblyline.googlecode.com

2 http://cpc2.cbi.pku.edu.cn/

3 http://metascape.org/

4 https://cran.r-project.org/

## Abbreviations

CCK8: Cell Counting Kit-8
DE: differentially expressed
ECM: extracellular matrix
FC: fold change
H/N: hypoxia/normoxia
HIFs: hypoxia-inducible factors
iWAT: inguinal WAT
LDH: Lactate dehydrogenase
LncRNAs: Long non-coding RNAs
OD: optical density
ORO: Oil Red O
qRT-PCR: quantitative RT-PCR
SD: standard deviation
TPM: transcripts per kilobase million
WAT: White adipose tissue.
